# Preoperative Progressive Pneumoperitoneum Revisited

**DOI:** 10.3389/fsurg.2021.754543

**Published:** 2021-10-18

**Authors:** Kristen E. Elstner, Yusuf Moollan, Emily Chen, Anita S. W. Jacombs, Omar Rodriguez-Acevedo, Nabeel Ibrahim, Kevin Ho-Shon, John Magnussen, John W. Read

**Affiliations:** ^1^Department of Surgery, Macquarie University Hospital, Macquarie University, Sydney, NSW, Australia; ^2^Hernia Institute Australia, Edgecliff, NSW, Australia; ^3^Department of Surgery, Royal Brisbane and Women's Hospital, Herston, QLD, Australia; ^4^Macquarie Medical Imaging, Macquarie University Hospital, Macquarie University, Sydney, NSW, Australia

**Keywords:** Botulinum Toxin A, loss of domain, incisional hernia, complex hernia, preoperative progressive pneumoperitoneum

## Abstract

Incisional hernia represents a common and potentially serious complication of open abdominal surgery, with up to 20% of all patients undergoing laparotomy subsequently developing an incisional hernia. This incidence increases to as much as 35% for laparotomies performed in high-risk patients and emergency procedures. A rarely used technique for enabling closure of large ventral hernias with loss of domain is *preoperative progressive pneumoperitoneum* (PPP), which uses intermittent insufflation to gradually stretch the contracted abdominal wall muscles, increasing the capacity of the abdominal cavity and allowing viscera to re-establish right of domain. This assists in tension-free closure of giant hernias which may otherwise be considered inoperable. This technique may be used on its own, or in conjunction with preoperative Botulinum Toxin A to confer paralysis to the lateral oblique muscles. These two complementary techniques, are changing the way complex hernias are managed.

## Introduction

Complex abdominal wall hernia is a challenging surgical condition, plagued by high recurrence rates. The comparatively recent introduction of chemical component relaxation using Botulinum Toxin A (BTA), as an adjunct in complex hernia surgery, has changed the parameters of what is feasible to repair. BTA injected preoperatively into the lateral oblique muscles provides temporary flaccid paralysis to the abdominal musculature and facilitates fascial closure of the defect at time of surgery, while maintaining the integrity of the abdominal wall tissues and surgical planes. Compounding the benefits of BTA is the addition of the longstanding, but underappreciated, technique of Preoperative Progressive Pneumoperitoneum (PPP), whereby intermittent insufflation of the peritoneal cavity is used to elongate the contracted oblique muscles and increase the capacity of the peritoneal cavity to hold viscera. PPP is a proven technique to facilitate repair of these large incisional hernias, particularly those with significant loss of domain.

## Methods

**Figure 1:**
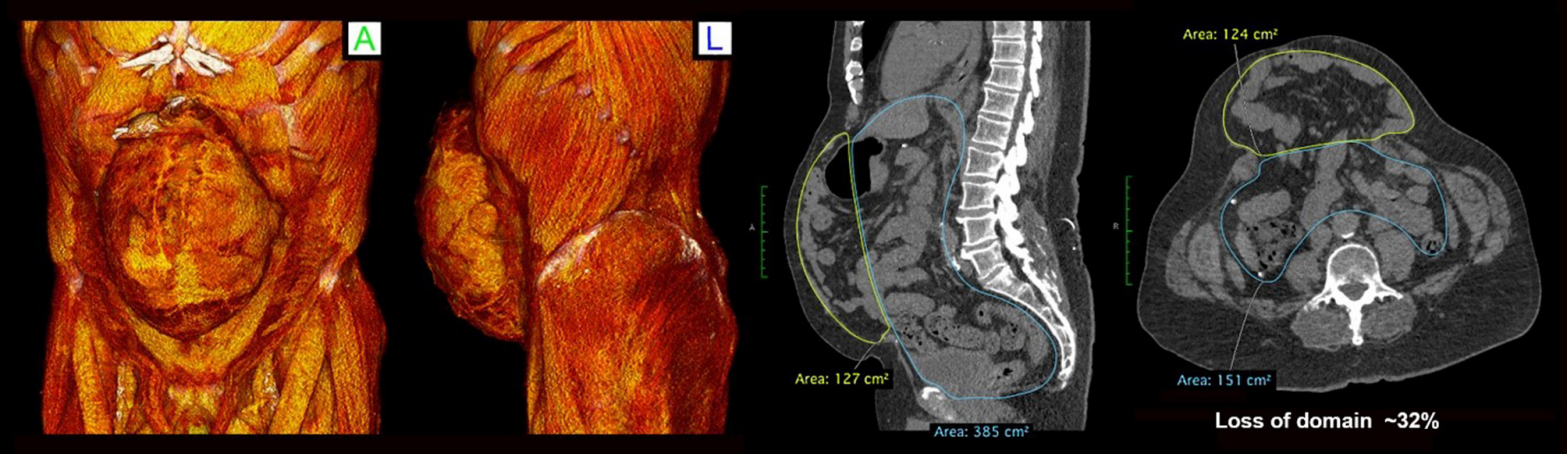
Baseline CT images demonstrating large incisional hernia post 2 open AAA repairs and two subsequent open incisional hernia repairs. Loss of domain is calculated at 32%.

Thirty-nine consecutive patients have been analyzed retrospectively from a prospectively maintained database between 2013–2021. All patients presented electively with large ventral incisional hernias, and were considered for PPP if their hernia was associated with loss of domain ≥15% and/or difficult laparoscopic access to the peritoneal cavity was anticipated. All patients underwent baseline abdominal wall CT imaging prior to BTA and PPP to measure their hernial defect size, loss of domain, and to assess the lateral abdominal wall length (Figure 1). These patients then underwent chemical relaxation injections with BTA at least 2 weeks preoperatively, and underwent a second CT scan to assess BTA effect. Five days prior to surgery, the patients underwent PPP catheter insertion, with insufflation over subsequent 5 days. Patients were then reassessed with a third CT scan the day prior to surgery.

### BTA Technique

This technique has been described in detail previously (1, 2).

The 39 patients underwent BTA infiltration as an outpatient procedure under ultrasound guidance. The patient was placed in a lateral position with the arm extended above the head and the leg straightened, increasing the distance between the anterior superior iliac spine and the lower coastal margin. The three layers of the lateral abdominal wall (external oblique, internal oblique, transversus abdominis) were identified using ultrasound and injection points marked (three sites on each side of the abdomen, as per Smoot et al. (4).

Earlier in the study period, BTA injections were performed to all 3 layers of the abdominal wall. From June 2016 onwards, the transversus abdominis layer was spared and only the external and internal oblique muscles underwent BTA injection (3).

Under aseptic technique, a total of 500 U of Dysport® (equivalent to 200 U of Botox®) was injected to the appropriate muscle bellies at the 6 previously identified sites.

### PPP Catheter Insertion Technique

**Figure 2:**
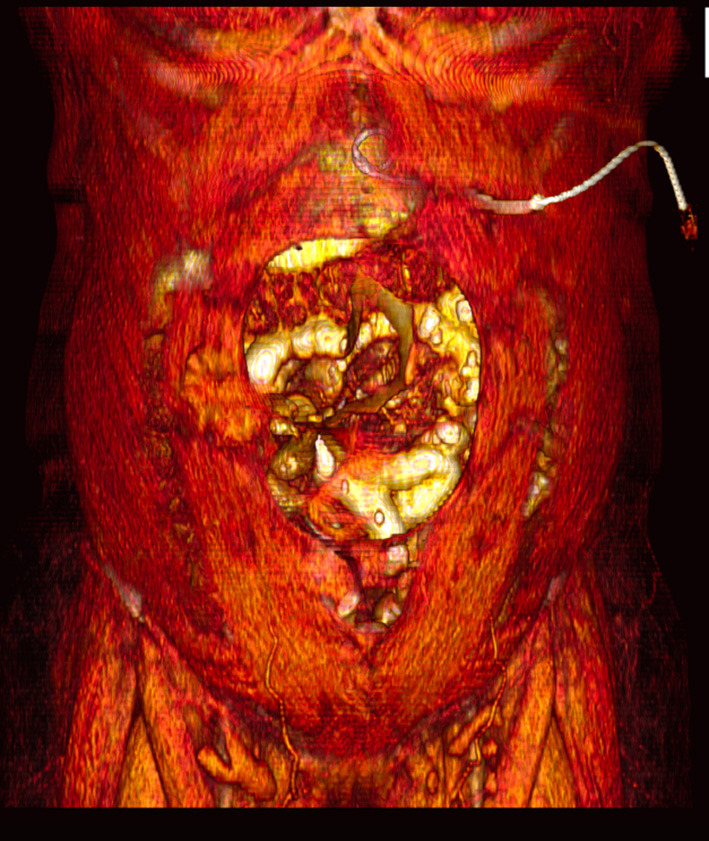
Three-Dimensional volume rendered CT image, demonstrating site of pigtail catheter insertion.

The PPP catheter was inserted by an interventional radiologist under ultrasound or CT guidance. Via an anterolateral approach, at an area remote from any scarring and incisions (Figure 2), a 22G spinal needle was inserted into the peritoneal cavity, such that the tip just crosses the peritoneal space. Three hundred ml of unfiltered room air was introduced via a syringe following which a confirmatory CT was performed. The largest pocket of intraperitoneal air was identified, and an 8Fr pigtail catheter was inserted into that pocket of air. Progressive insufflation continued daily or second daily on either an inpatient or outpatient basis, until the day of operation, using a 3-way valve. The volume of insufflation was on average 800–1000 ml per day, and dictated by patient tolerance. The level of abdominal wall distension and discomfort along with dyspnoea was used as indicators to cease insufflation.

During the PPP phase, patients were given daily enoxaparin injections (0.5 mg/kg) for venous thromboembolism prophylaxis. On the day prior to operation, a CT scan of the abdomen was performed at the same vertebral level as previous CT scans, and measurements of the abdominal wall length were recorded from the lateral edge of the quadratus lumborum to the medial edge of the rectus abdominus muscle.

### Operative Technique

At elective laparoscopic hernia repair, the pigtail catheter is initially connected to the carbon dioxide gas insufflator to establish pneumoperitoneum, following which operating ports are inserted as required. The surgical procedure consists of adhesiolysis with reduction of hernial content into the peritoneal cavity, defining the edges of the fascial defect, and reduction of the hernial sac. The hernial defect was closed using non-absorbable sutures (5 Ticron) in an interrupted shoelace fashion. IPOM mesh hernioplasty was undertaken using a composite polypropylene-seprafilm mesh (Ventralight™ ST, Ventralight™ ST-ECHO, Ventrio™ ST) secured using trans-fascial nylon sutures at the edges of the mesh and an absorbable strap fixation device. This has been described in detail previously (1).

Twenty-six (68.4%) patients required a minimally invasive external oblique release using micro-air blade device under vision (1). Post-operatively, patients were required to wear an abdominal binder and received DVT prophylaxis with enoxaparin during their hospital stay, and prophylactic IV antibiotics for 72 h. Early mobilization was encouraged.

## Results

**Figure 3:**
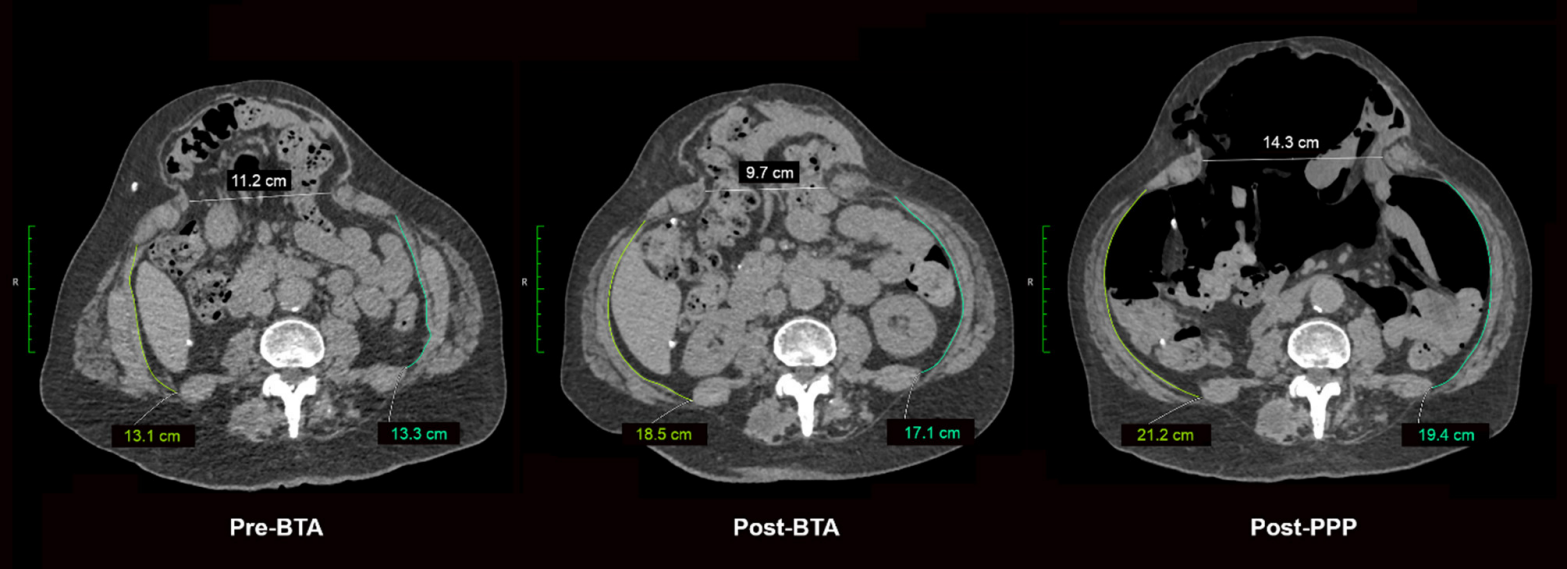
Axial CT images demonstrating progressive changes in abdominal circumference and hernia defect measurement changes (Baseline/Pre-BTA, Post-BTA and Post PPP). Measurements taken from the same vertebral level on each occasion.

Between 2013–2021, 39 patients underwent our protocol using BTA and PPP (Table 1). This cohort consisted of 17 females (43.6%) and 22 males (56.4%) with a mean age of 64 years (range 24–78 years) and mean BMI of 34.2 kg/m^2^ (range 21.8–58.7 kg/m^2^). The mean transverse defect size was 16.1 cm (range 7–25 cm) and mean loss of domain was 37.1% (range 12 to 67%). Twenty-six (68.4%) patients had at least one or more previous hernia repairs. All patients were required to cease smoking in the preoperative months, although one patient was found later to have continued smoking. The median time between BTA injection and PPP catheter insertion was 23 days. The mean time between PPP catheter insertion and operative repair was 5.7 days (SE ± 0.5 days, range 2 days to 16 days). Serial non-contrast CT imaging demonstrated a mean length gain of the lateral abdominal oblique after BTA of 3.2 cm/side (range 0.18–14.0 cm), which then increased to 4.4 cm/side (range 0.23–13.4 cm) after PPP (Figure 3). This gain was statistically significant (*t* = 1.68, *p* < 0.001).

**Table 1:** Demographic of patients undergoing preoperative PPP prior to repair of complex ventral hernia.

**Gender**	**n (%)**
Female	15 (39.5%)
Male	23 (60.5%)
**Age**	
Median (±SE)	63 year 10 months (±19 months)
Range	42–78 year 10 months
**BMI**	
Mean (±SE)	34.1 (±1.5)
Range	21.7–67.9
Obese (> 30.0)	24 (63.2%)
Obese (Class 1: 30.0–34.9)	11 (28.9%)
Obese (Class 2: 35.0–39.9)	8 (21.1%)
Morbidly Obese (Class 3: 40.0–49.9)	2 (5.3%)
Super Obese (> 50.0)	3 (7.9%)
**Cardiovascular**	
-Hypertension	10 (26.3%)
-IHD/AMI	3 (7.9%)
-AF	4 (10.5%)
-Other cardiovascular	1 (2.6%)
**Respiratory**	
-COPD	2 (5.3%)
-Smoker (within 3 months)	1 (2.6%)
-Ex-smokers (> 3 months)	15 (39.5%)
-OSA	1 (2.6%)
**Other Comorbidities**	
Diabetes mellitus	12 (31.6%)
Malignancy	4 (10.5%)

There were no complications associated with BTA injections. Twenty-three patients (60.5%) developed PPP—related complications, which included subcutaneous emphysema, pneumothorax, pneumomediastinum, pneumocardium and metabolic acidosis. Those complications were incidental findings, none required intervention, and resolved with deflation of the pneumoperitoneum.

Complete fascial closure and mesh hernia repair were performed in all patients using techniques described above, and which are described in further detail in previous publications (1). Intra-abdominal pressures were not formally measured, however, there were no instances of clinically apparent abdominal hypertension.

Post operatively, one patient developed a superficial wound infection which was managed conservatively with oral antibiotics. Two patients developed post-operative seroma requiring percutaneous aspiration, and one patient developed a port-site hernia diagnosed using post-operative imaging. There has not been any ventral hernia recurrence, with a median follow-up of 34 months (range 6 to 100 months).

## Discussion

Goni Moreno's preoperative pneumoperitoneum technique was first described over 70 years ago. Its initial description involved intermittent injections of intraperitoneal oxygen to preoperatively reduce an incarcerated epigastric hernia and was soon being used by Moreno in the preoperative management of large ventral hernias. This technique has been modified many times over subsequent decades, but the original tenets remain the same: inducing a pneumoperitoneum prior to surgery to increase right of domain of abdominal viscera.

PPP is indicated when it is not possible to perform primary fascial closure due to the size of the hernia and/or loss of domain; or when sudden reintroduction of the hernia into the peritoneal space would result in disastrous consequences such as abdominal compartment syndrome. A commonly reported hernia/abdominal volume of >15–20% is predictive of a problematic closure (5–7). Less commonly reported indications are the adhesiolytic effect of the insufflation potentially reducing operative time; and allowing for visualization (on CT imaging) of safe areas for initial port placement (2).

The available evidence in the literature is limited with regards to critical evaluation of the PPP technique, specifically the optimal duration, frequency of insufflation, or volume of insufflation (8). Anecdotal reports and smaller studies, scattered throughout the literature, are quite variable. A 2016 review on tissue expander techniques identified only 15 PPP studies involving 269 patients over a 75-year period (9). The length of time for insufflation reported over many years in the literature ranges from 3 to 100 days. In more modern times, a 2020 paper reporting on 162 PPP cases, used on its own as a pre-operative adjunct reported a median insufflation time of 15 days (10). As highlighted in Alam's 2016 systematic review, (8, 9) a tension-free fascial closure was achieved in 84% of cases, with a reported recurrence rate of 7.2% when PPP is used as a stand-alone adjunct.

What has changed within the past decade is the addition of Botulinum Toxin A in the management of complex ventral hernias, and its application in conjunction with PPP presents an interesting development. BTA is administered prior to PPP and allowed to achieve maximal paralysis. When the two techniques are combined, the need for prolonged insufflation is negated, and subsequently, PPP insufflation need only be performed for a few days prior to surgery. Bueno-Lledo et al. (11, 12) have reported the largest cohort of patients treated with BTA and PPP prior to surgery for large incisional hernia. Eighty patients who presented electively with large incisional hernias with volume of incisional hernia/volume of abdominal cavity (VIH/VAC) ratio > 20% were given BTA at 40 days pre-operatively, and then PPP at 14 days preoperatively. This was independent of size defect measurements. Complete fascial primary closure was seen in 77 patients (96.7%), with mesh bridging in three cases. At a mean follow-up of 38.5 months, there were five hernia recurrences (6.2%). They noted that, using BTA and PPP, they achieved a mean decrease of 16.3% in the VIH/VAC ratio, allowing the reintroduction of the herniated viscera into the peritoneal cavity.

## Conclusion

Preoperative adjuncts BTA and PPP have added new capabilities in the repair of complex ventral hernia, especially when used in conjunction. Although each have their own indications, advantages and disadvantages, they are complimentary to one another. PPP should be considered in large recurrent hernias and those with significant loss of domain.

## Data Availability Statement

The raw data supporting the conclusions of this article will be made available by the authors, without undue reservation.

## Author Contributions

Radiology advice and images provided by JR, KH-S, and JM. Project co-ordination and oversight NI. All authors contributed to the development of the project, manuscript, and written work. All authors edited the written work. All authors contributed to the article and approved the submitted version.

## Conflict of Interest

The authors declare that the research was conducted in the absence of any commercial or financial relationships that could be construed as a potential conflict of interest.

## Publisher's Note

All claims expressed in this article are solely those of the authors and do not necessarily represent those of their affiliated organizations, or those of the publisher, the editors and the reviewers. Any product that may be evaluated in this article, or claim that may be made by its manufacturer, is not guaranteed or endorsed by the publisher.
